# Decisions to Start, Strengthen, and Sustain Food Fortification Programs: An Application of the Grading of Recommendations Assessment, Development, and Evaluation (GRADE) Evidence to Decision (EtD) Framework in Nigeria

**DOI:** 10.1093/cdn/nzac010

**Published:** 2022-01-31

**Authors:** Valerie M Friesen, Mduduzi N N Mbuya, Frank T Wieringa, Chito N Nelson, Michael Ojo, Lynnette M Neufeld

**Affiliations:** Knowledge Leadership, Global Alliance for Improved Nutrition (GAIN), Geneva, Switzerland; Food, Nutrition, Health, UMR QualiSud, French National Research Institute for Sustainable Development (IRD), Montpellier, France; UMR QualiSud, University of Montpellier, Avignon University, CIRAD, Institut Agro, French National Research Institute for Sustainable Development (IRD), University of Réunion, Montpellier, France; Knowledge Leadership, Global Alliance for Improved Nutrition (GAIN), Washington, DC, USA; Food, Nutrition, Health, UMR QualiSud, French National Research Institute for Sustainable Development (IRD), Montpellier, France; UMR QualiSud, University of Montpellier, Avignon University, CIRAD, Institut Agro, French National Research Institute for Sustainable Development (IRD), University of Réunion, Montpellier, France; Food and Nutrition Division, Department of Social Development, Ministry of Budget and National Planning, Abuja, Nigeria; Global Alliance for Improved Nutrition (GAIN), Abuja, Nigeria; Knowledge Leadership, Global Alliance for Improved Nutrition (GAIN), Geneva, Switzerland

**Keywords:** decision making, food fortification, GRADE, Evidence to Decision, recommendations, Nigeria

## Abstract

**Background:**

Although the potential impact of food fortification to improve the micronutrient status of populations has been demonstrated beyond a doubt, it is constrained in practice by critical gaps in program design and implementation. These are partly linked to suboptimal decision making.

**Objectives:**

We aimed to demonstrate how the Grading of Recommendations Assessment, Development, and Evaluation (GRADE) Evidence to Decision (EtD) framework for health system and public health decisions can be applied to formulate recommendations and make decisions in national food fortification programming.

**Methods:**

Following a program impact pathway, we reviewed the literature to define the key decision types and identify the corresponding decision makers necessary for designing and implementing effective food fortification programs. We then applied the GRADE EtD framework to the Nigerian fortification program to illustrate how evidence-informed assessments and conclusions can be made.

**Results:**

Fortification program decisions were classified into 5 types: *1*) program initiation; *2*) program design; *3*) program delivery; *4*) program impact; and *5*) program continuation. Policymakers, food processors, and (in cases dependent on or considering external funding) development partners are the main decision makers in a fortification program, whereas technical partners play important roles in translating evidence into contextualized recommendations. The availability and certainty of evidence for fortification programs are often low (e.g., quality and coverage data are not routinely collected and there are challenges evaluating impact in such population-based programs using randomized controlled trials) yet decisions must still be made, underscoring the importance of using available evidence. Furthermore, when making program initiation and continuation decisions, coordination with overlapping micronutrient interventions is needed where they coexist.

**Conclusions:**

This framework is a practical tool to strengthen decision-making processes in fortification programs. Using evidence in a systematic and transparent way for decision making can improve fortification program design, delivery, and ultimately health impacts.

## Teaser

This article demonstrates how a decision-making framework for health system and public health decisions can be applied to national food fortification programs.

## Introduction

Although the potential impact of food fortification to improve the micronutrient status of populations has been demonstrated beyond a doubt (1), there are critical gaps in how programs are designed and implemented. This manifests in what has been termed an unfinished agenda, which can be summarized as follows: first, not all countries that could benefit from fortification have mandatory or voluntary programs in place; and second, where programs are in place, many countries are not reaching their potential for impact owing to large gaps in quality (i.e., low coverage of fortified foods generally and even lower coverage of fortified foods that meet national fortification standards), inequity (i.e., fortified foods are not available and/or affordable for the poorest segments of the population), and feasibility [i.e., low coverage of industrially processed (i.e., fortifiable) foods] (2). Despite these issues, fortified foods have been shown to be major contributors to intakes of key micronutrients, such as vitamin A, iodine, and folic acid, in many countries (3–5) and several program evaluations have demonstrated impact on biological outcomes [e.g., goiter (6), neural tube defects (7), and anemia (8)]. In addition to unrealized potential, if the aforementioned quality gaps are fully addressed, there may be a risk of excessive micronutrient intakes in some settings (3). Such risks and their concomitant effects on individual and population health (9, 10) are of particular concern because they may be exacerbated in contexts of cumulative micronutrient intakes from fortified foods plus other dietary sources and/or micronutrient interventions (e.g., supplementation).

These design and implementation challenges can be partly linked to suboptimal decision making. Results from fortification coverage surveys of edible oil, wheat flour, and maize flour in 8 countries identified 2 primary issues related to low coverage (11). First, poor choice of food for fortification (i.e., the food selected was not a staple or was predominately consumed in a nonfortifiable form), which is a program design issue. For example, a high proportion of households consumed maize flour in Tanzania (93%), Uganda (92%), and Nigeria (Kano state) (77%), but only 37%, 42%, and 11%, respectively, consumed it in a fortifiable form. Second, in several countries food processors are failing to fortify owing to poor monitoring and enforcement of fortification mandates and/or lack of incentives for industry to fortify, which is a program delivery issue. This issue cut across all food vehicles assessed.

Although part of the problem is the limited availability of evidence to inform program decisions (12), a more pertinent issue is the absence of an explicitly articulated framework that structures the fortification program cycle and identifies key decisions to be made at varying stages. Decision-making frameworks have been used extensively for improving the quality of health care (e.g., clinical recommendations, coverage decisions, and decisions about diagnostic tests) (13) and further adapted for use in making health system and public health decisions (14). Such frameworks provide a systematic and transparent process for formulating evidence-informed recommendations and making decisions at critical junctures, with an emphasis on consideration and documentation of all important criteria and the use of the best available evidence.

In this article, we demonstrate how a decision-making framework for health system and public health decisions can be applied to formulate recommendations and make decisions in national large-scale food fortification programs and illustrate the process using a real-world example from Nigeria.

## Methods

### Defining decision types and decision makers for food fortification programs

Following the program impact pathway (PIP) for large-scale food fortification as put forth by Martorell et al. (8), we reviewed the literature to define the key decision types and identify the corresponding decision makers necessary for designing and implementing effective large-scale food fortification programs. The PIP illustrates with specificity the underlying program theory (i.e., how a program is envisaged to work). Importantly, it outlines the sequentially dependent program steps and linkages. As such, it can be used to illustrate critical assumptions and necessary processes and inform the decisions that are required at each step to make the program work.

### Selection and description of the decision-making framework

We selected the Grading of Recommendations Assessment, Development, and Evaluation (GRADE) Evidence to Decision (EtD) framework for health system and public health decisions (14) based on its relevance to large-scale food fortification as a population-based public health program and the global acceptance and use of GRADE EtD frameworks by >100 organizations worldwide, including the WHO and the Cochrane Collaboration (13). The GRADE EtD framework consists of 3 main steps: *1*) formulating the question; *2*) making an informed assessment; and *3*) drawing conclusions. In the first step, a general description of the problem and the question details are defined [i.e., problem, option, comparison, main outcomes, setting, perspective from which the decision is being made (population or individual), subgroups, and background]. In the second step, data sources are identified, specific criteria are assessed (i.e., priority of the problem, benefits and harms, values, balance of effects, resources required, equity, acceptability, and feasibility), and a judgment for each criterion is made (**Table 1**) (14). In the third step, a summary of the judgments for all criteria is made followed by a recommendation. The strength of the recommendation is defined such that a strong recommendation indicates the panel is confident that the benefits outweigh the harms, whereas a conditional recommendation indicates that the panel is less confident and therefore also includes specific guidance on the conditions required for implementing it. Finally, a detailed justification summarizing the most important criteria is provided along with any necessary considerations related to subgroups, implementation, monitoring, evaluation, and research priorities.

**TABLE 1:** Criteria and judgments in the Grading of Recommendations Assessment, Development, and Evaluation (GRADE) Evidence to Decision (EtD) framework for health system and public health recommendations^1^

Criterion	Judgment options							
Priority of the problem	Is the problem a priority?	Don't know	Varies	—	No	Probably no	Probably yes	Yes
Benefits and harms	How substantial are the desirable anticipated effects?	Don't know	Varies	—	Trivial	Small	Moderate	Large
	How substantial are the undesirable anticipated effects?	Don't know	Varies	—	Large	Moderate	Small	Trivial
Certainty of the evidence of effects	What is the overall certainty of the evidence of effects?	No included studies	—	—	Very low	Low	Moderate	High
Values	Is there important uncertainty about or variability in how much people value the main outcomes?	—	—	—	Important uncertainty or variability	Possibly important uncertainty or variability	Probably no uncertainty or variability	No important uncertainty or variability
Balance of effects	Does the balance between desirable and undesirable effects favor the option or the comparison?	Don't know	Varies	Favors the comparison	Probably favors the comparison	Does not favor either the option or the comparison	Probably favors the option	Favors the option
Resources required	How large are the resource requirements (costs)?	Don't know	Varies	Large costs	Moderate costs	Negligible costs or savings	Moderate savings	Large savings
Certainty of evidence of resources required	What is the certainty of the evidence of resource requirements (costs)?	No included studies	—	—	Very low	Low	Moderate	High
Cost-effectiveness	Does the cost-effectiveness of the option (the out-of-pocket cost relative to the net benefits) favor the option or the comparison?	Don't know	Varies	Favors the comparison	Probably favors the comparison	Does not favor either the option or the comparison	Probably favors the option	Favors the option
Equity	What would be the impact on health equity?	Don't know	Varies	Reduced	Probably reduced	Probably no impact	Probably increased	Increased
Acceptability	Is the option acceptable to key stakeholders?	Don't know	Varies	—	No	Probably no	Probably yes	Yes
Feasibility	Is the option feasible to implement?	Don't know	Varies	—	No	Probably no	Probably yes	Yes

1 Adapted from Moberg et al. (14) under the terms of the Creative Commons Attribution 4.0 International License (http://creativecommons.org/licenses/by/4.0/).

### Applying the decision-making framework to food fortification program decisions

We applied the GRADE EtD framework to a real-world example, namely a recommendation regarding modifying the design of the large-scale food fortification program in Nigeria to reduce the risk of excessive vitamin A intakes, using the interactive EtD tool template for health system and public health decisions (https://ietd.epistemonikos.org). The completed EtD framework was then reviewed and interpreted by a panel comprised of the authors and a small group of stakeholders from governmental organizations involved in Nigeria's national food fortification program in a virtual workshop, followed by email communications to provide further detail and assessments. This was not a full panel of all relevant stakeholders in Nigeria, however, because that was beyond the scope of the current work; therefore, judgments and conclusions are our own. The methods deployed in this study primarily involved a narrative review of the literature, coupled with an analysis of data extracted from published articles. As such, they did not meet the definition of research with human subjects and, consequently, were not submitted for ethical review.

## Results

### Decision types and decision makers for food fortification programs

**FIGURE 1:**
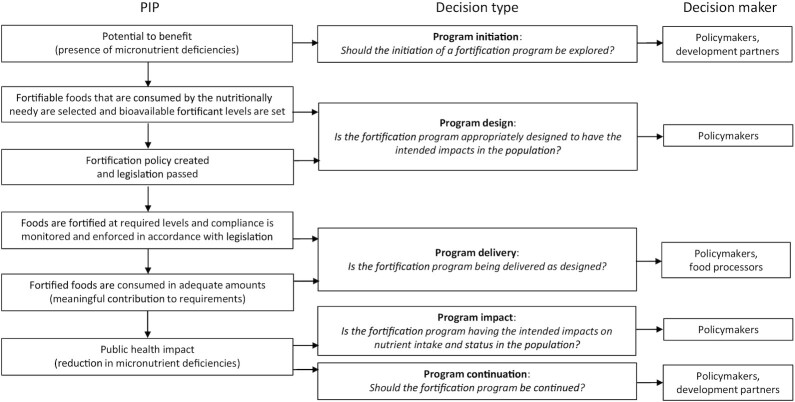
Large-scale food fortification program decision types and decision makers mapped to the PIP [adapted from Martorell et al. (8) under the terms and conditions for articles published under the ASN free access publishing option (http://www.nutrition.org/publications/guidelinesand-policies/license/)]. PIP, program impact pathway.

The main fortification decisions were classified into the following 5 decision types mapped to the PIP for large-scale food fortification programs: *1*) program initiation; *2*) program design; *3*) program delivery; *4*) program impact; and *5*) program continuation (**Figure 1**). First is the demonstrated need for food fortification in the population. The prevalence of micronutrient deficiencies suggests the potential to benefit from micronutrient interventions, such as fortification, which informs the decision of whether to explore the initiation of a fortification program as a strategy to address the identified deficiencies. Second, if a program is justified, the magnitude of that need and the consumption patterns of the potentially fortifiable foods as well as other micronutrient sources (in the diet and from other interventions) in the population are critical to inform key decisions related to program design, such as the selection of the staple foods to fortify and the type and amount of fortificants to add (which are then defined in relevant fortification policies and legislation), to ensure the program is appropriately designed to have the intended impacts in the population. Third, once a program has begun implementation, the quality (i.e., compliance with national fortification standards), coverage, and consumption of fortified foods in the population (and subgroups) inform decisions related to program delivery because they provide evidence to understand how well the program is performing relative to its design and whether the fortified foods are making meaningful contributions to micronutrient intakes. Finally, the change (or lack thereof) in micronutrient deficiencies in the population (and subgroups) informs decisions related to the public health impact of the program and the continued need for the program over time. The latter decision type in addition requires consideration of factors external to the fortification program, such as changes in the availability and consumption of other micronutrient-rich foods and coverage of other overlapping micronutrient interventions in the population (and subgroups).

Policymakers (particularly government ministries) and food processors are the main decision makers in a fortification program (Figure 1). Policymakers are key stakeholders for all 5 decision types given their responsibility for developing and implementing policies to protect and improve population health, which includes supporting food fortification programs in various capacities (**Table 2**). Food processors are relevant for program delivery decisions given they are responsible for ensuring the presence of fortificants in the foods selected for fortification in amounts stated in the national fortification standards. Development partners (e.g., bilateral donors, multilateral agencies, and private foundations) are also relevant for program initiation and continuation decisions in contexts dependent on external funding, where such funding is under consideration, and/or where technical assistance is needed. Finally, other technical partners (e.g., nongovernmental organizations and the research community) play important roles in translating data and evidence into contextualized recommendations that meet the needs of different decision makers for all decision types.

**TABLE 2:** Typical policymakers and their decision-making roles in national food fortification programs

Typical policymaker	Role in program
Ministry of Health	Make decisions related to the formulation and implementation of fortification policies and legislation
Ministry of Finance (or Budget and Planning Commission)	Make decisions related to the allocation of funds to support fortification program design, implementation, monitoring, and evaluation
Standards authorities	Make decisions related to the development of fortification standards
Regulatory and food control authorities	Make decisions related to the enforcement of fortification legislation and standards

### Application of the GRADE EtD framework to food fortification programs

#### Formulating the question

In the example framework in **Supplemental File 1**, the question was formulated as, “Should the design of Nigeria's large-scale food fortification program, which aims to reduce vitamin A deficiency, be modified to reduce the risk of excessive vitamin A intakes?” (i.e., a program design decision). The problem was defined as a goal of reducing vitamin A deficiency in Nigeria through large-scale food fortification without exceeding the tolerable upper intake level (UL) for vitamin A intake in any subgroup of the population. The option considered in the framework was to modify the design of the fortification program by updating the selection of foods to be fortified with vitamin A and/or amounts of vitamin A to be added based on recent data on population need and consumption patterns. The comparison was to continue implementation of the fortification program as currently mandated [i.e., fortification of oil; sugar; and wheat, semolina, and maize flours with vitamin A as per current national standards (15–19)]. The main outcomes considered were vitamin A deficiency prevalence, vitamin A intakes from all dietary sources, and vitamin A intakes from fortified foods alone. The decision setting was a national recommendation for Nigeria from a population-level perspective. Although large-scale food fortification is a population-based program that does not target specific population groups, 2 subgroups were considered in making the recommendation, i.e., women of reproductive age (15–49 y old) and children (<5 y old), because they are the most at risk of micronutrient deficiencies and often the focus of fortification program design, monitoring, and evaluation efforts.

#### Making an assessment

The following is a summary of the research evidence, additional considerations, and judgments for each criterion assessed.

##### Priority of the problem

The problem was reducing vitamin A deficiency through large-scale food fortification in Nigeria without exceeding the UL for vitamin A intake in any subgroup of the population. In the most recent national micronutrient survey conducted in Nigeria in 2001, 30% of children <5 y old had vitamin A deficiency (serum retinol concentration <20 µg/dL) and 13% of mothers and 19% of pregnant women were at risk of vitamin A deficiency (<30 µg/dL), of whom 4% and 9%, respectively, were deficient (<20 µg/dL) (20). To increase vitamin A intakes in the population, several interventions are currently in place, including mandatory fortification of 5 staple foods with vitamin A, routine public health supplementation among children 6–59 mo of age, point-of-use fortification, biofortification, promotion of dietary diversity, voluntary fortification (e.g., infant formula, powdered milk, and cocoa drinks), and ad hoc individual supplement use (21). Unfortunately, there are no more recent national data available on the prevalence of vitamin A deficiency (nor adequacy of intakes) to demonstrate the extent to which the introduction of these programs has led to reduced deficiency prevalence (or increased intakes). At the same time, concerns have been raised in Nigeria regarding the risk of potentially excessive micronutrient intakes because of these multiple overlapping interventions that target the same micronutrient and similar population groups (21). For preformed vitamin A in particular, the effects of chronic excessive intakes can lead to toxicity, which may cause severe adverse effects (e.g., liver damage, teratogenicity) (22). It is therefore crucial to ensure that the total vitamin A intake in the diet coming from all sources does not result in intakes routinely exceeding the UL, yet is still enough to shift inadequate intakes to adequate. Based on this, the panel's judgment was that the problem of reducing vitamin A deficiency through large-scale food fortification without exceeding the UL for vitamin A intake in any subgroup of the population is probably a priority.

##### Benefits and harms, values, and balance of effects

The panel determined that the desirable effects of modifying the design of the fortification program by updating the selection of foods to fortify with vitamin A and/or amounts of vitamin A to be added are moderate, the undesirable effects are trivial, and the overall certainty of effects is very low. There were no studies comparing the option with the comparison; however, there were relevant findings from 1 cross-sectional subnational study that reported apparent vitamin A intakes from fortified foods alone based on actual consumption patterns (3). In that study, it was estimated that apparent vitamin A intakes would exceed the UL in 18% and 56% of women of reproductive age in Lagos and Kano states, respectively, if all foods were fortified according to national standards. Total vitamin A intakes are expected to be higher when intakes from all sources are considered (21). That said, currently compliance with mandatory fortification standards has been shown to be consistently poor with most foods (apart from salt) being fortified below standards or not at all (11, 23, 24) and coverage of other vitamin A interventions, such as supplementation and promotion of dietary diversity, is similarly low [e.g., in 2018, vitamin A supplementation reached only 41% of children 6–23 mo old nationally (25) and, in 2013, only 52% of children 6–23 mo old reported having consumed vitamin A–rich foods (26)]. This likely reduces the present risk of excessive vitamin A intakes in the population; however, if these programs were to improve and be implemented as intended, the risk would increase. There was no evidence on how the population values the main outcomes that were considered. Based on this, the panel's judgment was that there is probably no important uncertainty or variability in how much people value the main outcomes and that the balance of the desirable and undesirable effects probably favors modifying the design of the fortification program by updating the selection of foods to fortify with vitamin A and/or amounts of vitamin A to be added rather than continuing to implement it as currently mandated.

##### Resources required and cost-effectiveness

Costs incurred by food processors related to the fortification process and by government ministries related to monitoring the compliance of fortified foods with national standards must be considered along with costs required to implement other overlapping vitamin A interventions. Cost-effectiveness studies for the Nigeria fortification program specifically have not yet been done and the cumulative costs of all ongoing vitamin A interventions are unknown. However, it is assumed that if modifying the design of the fortification program by updating the selection of foods to fortify with vitamin A in Nigeria leads to fewer foods required to be fortified, then there would likely be some cost savings for both food processors and government ministries. Conversely, if new food vehicles are added, there may be additional costs. Optimizing the set of vitamin A interventions that can be effectively implemented in Nigeria to achieve the greatest impact would likely reduce cost inefficiencies of running multiple programs with low fidelity (compliance). Based on this, the panel's judgment was that, although there may be potential for moderate savings, they do not know the resources required nor the cost-effectiveness of the option as opposed to the comparison.

##### Equity

There is evidence from 2 cross-sectional surveys in 4 out of the 36 states of Nigeria (i.e., Kano, Lagos, Ebonyi, and Sokoto) that the coverage of the fortifiable foods currently mandated to be fortified with vitamin A is generally lower in vulnerable households, specifically those that are at risk of poverty (multidimensional poverty index ≥0.33), have low socioeconomic status (lowest 2 wealth quintiles), and have low dietary diversity (women's dietary diversity score <5 out of 10 food groups the previous day) (27, 28). Similar trends have been shown in other countries and may be due to issues of access, affordability, and/or limited consumption of the respective fortified foods among these at-risk groups (11). Comparatively, in Ebonyi and Sokoto, coverage of fortifiable bouillon (which is not currently included in the fortification program) was found to be universal (>98%) with no differences by vulnerable group (28). Although updating the selection of foods to fortify with vitamin A based on current consumption patterns would not change existing inequities related to access and affordability, the process would be able to identify which foods currently being fortified and which alternative and/or additional foods have the greatest potential to reach vulnerable populations. Based on this, the panel's judgment was that modifying the design of the fortification program by updating the selection of foods to fortify with vitamin A based on recent data on consumption patterns probably increases equity.

##### Acceptability

Fortification of staple foods in Nigeria is assumed to be generally accepted by the population because it neither changes the characteristics of the food nor requires any changes to consumption patterns, but no published studies are available to confirm this. Other key stakeholders, including policymakers, food processors, and development partners, have publicly demonstrated their support for the national fortification program in its current form while recognizing the need for improvements if reductions in micronutrient deficiencies are to be realized (29–31). Therefore, because these stakeholders already accept the current program, it is assumed that they would accept the option of a modified program that would better achieve its goal of reducing vitamin A deficiency while minimizing any risks of excessive intakes and cost inefficiencies, but there is no evidence to confirm this. Based on this, the panel's judgment was that they do not know if updating the selection of foods and/or amounts of vitamin A in Nigeria's fortification program is acceptable to all stakeholders.

##### Feasibility

Modifying the design of the fortification program by updating the selection of foods to fortify with vitamin A and/or amounts of vitamin A to add would require recent population data on vitamin A intakes and consumption patterns of fortifiable foods. In addition, recent data on vitamin A status would be ideal to serve as a new baseline against which to evaluate program impact in the future. These data are forthcoming as part of the 2021 National Food Consumption and Micronutrient Survey (data are expected to be available in 2022). Technical support and related funding to analyze the new data and propose specific program design changes would also be needed, which may be potential barriers in Nigeria. Once redesigned, fortification standards would need to be updated along with any necessary policy and legislative changes, which would require government buy-in and may be a possible barrier if stakeholders are not accepting of the option or processes are lengthy. Beyond that, the option would be delivered through the existing fortification program structures and thus as likely to succeed as the comparison (i.e., the current program). Based on this, the panel's judgment was that modifying the design of the fortification program to reduce the risk of excessive vitamin A intakes by updating the selection of foods and/or amounts of vitamin A is probably feasible.

#### Drawing conclusions

##### Recommendation and justification

The panel made a conditional recommendation for modifying the design of Nigeria's fortification program to reduce the risk of excessive vitamin A intakes by updating the selection of foods to be fortified with vitamin A and/or amounts of vitamin A to be added based on recent data on population need and consumption patterns (**Box 1**). The justification for this decision was based on the balance between the desirable and undesirable effects and the probable impact on equity. The conditionality of recommending it was based on the need to first fill evidence gaps that were identified in the EtD framework, namely the forthcoming data on nutrient status, nutrient gaps, and food consumption patterns being collected in the 2021 National Food Consumption and Micronutrient Survey (data are expected to be available in 2022); resources required; cost-effectiveness; and acceptability among stakeholders.

BOX 1 **Conclusions from the Grading of Recommendations Assessment, Development, and Evaluation (GRADE) Evidence to Decision (EtD) framework for a decision about modifying the design of Nigeria's large-scale food fortification program to reduce the risk of excessive vitamin A intakes**

RECOMMENDATION: In Nigeria, modifying the design of the national food fortification program to reduce the risk of excessive vitamin A intakes in the population by updating the selection of foods to be fortified with vitamin A and/or amounts of vitamin A to be added based on recent data on population need and consumption patterns is conditionally recommended.

Remarks:

- The conditionality of this recommendation was based on the need to first fill evidence gaps that were identified in the EtD framework, namely resources required, cost-effectiveness, and acceptability among stakeholders, which should be immediate research priorities.
- The low certainty of the evidence comparing the desirable and undesirable effects should not be a barrier to adopting this recommendation given that high-certainty evidence studies (e.g., randomized controlled trials, systematic reviews) examining the option in opposition to the comparison are neither feasible nor necessary to undertake in the context of a national population-based program for which efficacy of the intervention has already been demonstrated in such studies, as is the case for vitamin A fortification.
- The modifications should be based on the demonstrated vitamin A needs and consumption patterns of fortifiable foods among different subpopulation groups (e.g., children <5 y old, adolescent boys and girls, women of reproductive age, and adult men) following global guidance on designing fortification programs and using data that are forthcoming from the 2021 National Food Consumption and Micronutrient Survey.
- Implementation of this recommendation should be subject to ongoing monitoring to ensure high-quality implementation according to its design, including:
  - compliance monitoring at import, production, and market levels with effective enforcement measures as relevant; and
  - coverage and consumption monitoring at household and individual levels as relevant.
- Monitoring of vitamin A intakes from fortified foods must be coordinated with that from other interventions that similarly aim to increase vitamin A intakes to ensure that the total vitamin A intake in the diet is considered when reviewing risks of excessive intakes.

## Discussion

In this article, we prepared the GRADE EtD framework for health system and public health decisions for a real-world example regarding a recommendation for modifying the design of the large-scale food fortification program in Nigeria to reduce the risk of excessive vitamin A intakes. The Nigeria example demonstrated the utility of the EtD framework to help policymakers guide and strengthen fortification program decision-making processes to ensure they are systematic, structured, and transparent. In addition, it highlighted the flexibility of its use because it does not require significant resources to populate and is still effective even when evidence is lacking, given the aim is simply to document the evidence (or lack of) that was used to make judgments.

Using an EtD framework alongside a PIP can help improve decision making in national food fortification programs. By defining the main fortification program decisions and mapping them to the PIP, the fortification program cycle is explicitly articulated and the end goal to be reached at each stage is clarified. This understanding is an important first step in strengthening decision-making processes because although fortification programs are intended to serve as medium- to long-term interventions to address micronutrient deficiencies (with dietary diversification being the ultimate long-term goal) (32), in reality they are often put in place with little to no review or planning for future adjustments (33). Using an EtD framework as the basis for in-depth review with fortification stakeholders can enhance engagement by organizing a large quantity of information into clear steps for review guided by a set of program-relevant questions and criteria. In addition, it can strengthen the credibility of decisions made by documenting the evidence in a systematic and transparent way and can increase uptake of findings by decision makers given its accessible format (14).

The Nigeria example in this article illuminates several key findings regarding fortification program decision making that are relevant across countries, as follows.

The availability and certainty of evidence for population-based public health interventions, such as fortification, are often low or very low, yet decisions must still be made, underscoring the importance of using the best available evidence (34). For decisions related to program initiation, impact, and continuation, the option and comparison in the EtD framework are essentially fortification compared with no fortification in the given setting. As such, there would likely be high-certainty evidence studies (such as systematic reviews) that demonstrate the efficacy of fortification (although not necessarily in the specific country where the program occurs). In addition, effectiveness studies that use variable study designs to evaluate the impact of fortification programs among populations in real-world settings are increasing given the challenges with evaluating population-based programs using traditional designs such as randomized controlled trials (35). On the other hand, for decisions related to program design and delivery, the likelihood of having such high-certainty evidence studies (such as randomized controlled trials) that compare different options for a specific national program is very low. In those cases, it may be necessary to rely on lower-certainty data sources, such as routine monitoring data for ongoing programs on quality and observational studies on coverage and consumption of fortified foods [although not consistently collected across countries (33), where available, monitoring data can be found in the Global Fortification Data Exchange (36)]. This was the case in the Nigeria example, which compared 2 different program design options and relied on evidence from 1 observational study that assessed only 1 of the main outcomes in the framework (i.e., vitamin A intakes from fortified foods alone) to examine the desirable and undesirable effects because this was the best available evidence despite its low certainty.

Fortification program decisions do not always occur in a linear process as shown in the PIP. Whereas at the onset of a program decisions are likely to proceed in order (i.e., initiation, design, delivery, and impact), evidence at delivery and impact stages may trigger the need to revisit previous program decisions downstream in the PIP. This was the case in the Nigeria example, where evidence from a study examining the potential for program impact triggered the need to consider a program design decision because it estimated a high risk of excessive vitamin A intakes if the program were implemented as currently designed, but with greater fidelity (compliance). Regular assessments of the quality of implementation and initial design assumptions (particularly as they relate to micronutrient needs and consumption patterns of fortifiable foods) are essential as part of monitoring a fortification program throughout the program life cycle (33). Although these periodic adjustments have long been recommended in global fortification guidelines (32), in practice few programs have adjusted their fortification standards after being initiated. One notable exception is fortification of sugar with vitamin A in Guatemala, where required amounts of vitamin A in fortification standards were lowered and vitamin A supplementation campaigns were revised to exclude certain child age groups in response to evidence of declining vitamin A deficiency prevalence and increasing vitamin A intakes over time (37).

Fortification decisions related to program initiation, design, and continuation should be made in coordination with those (decisions) that pertain to other complementary micronutrient deficiency control interventions where they coexist. Ideally, decisions on what set of programs should be implemented in a country should be made jointly by all stakeholders involved in micronutrient deficiency control interventions and optimized to maximize impact and cost-effectiveness while ensuring safety over time. However, currently these interventions are often initiated and implemented independently by different national and/or international stakeholders (38) and there is an absence of guidance or regulations to define how to effectively coordinate (9). This was the case in the Nigeria example where multiple vitamin A interventions were being implemented in isolation from one another and their cumulative contribution to vitamin A intakes was unknown. To identify the optimal set of vitamin A deficiency control interventions to achieve a desired level of effective coverage at the lowest cost, an optimization model was used in Cameroon (38). However, this method requires subnationally disaggregated data on micronutrient intakes and detailed costing information, along with considerable technical and financial resources to conduct the analyses, which are seldom available in many low- and middle-income countries without external resources. To improve coordination across programs, a national coordination body with strong leadership and a broad vision of nutrition has been suggested to promote balanced, safe, and impactful programs (10). Colombia is one such country that has striven to do this through the development of a separate commission whose purpose it is to strengthen the governance and coordination mechanisms of the different governing entities of their National Food and Nutrition Security Policy, which includes the national micronutrient deficiency prevention and control strategy (39).

There are some limitations to this article and the use of the GRADE EtD framework for fortification program decision making. First, the example framework was populated by the authors and a small group of stakeholders but did not include a full panel of all relevant stakeholders in Nigeria (including, but not limited to, those involved in fortification as well as other vitamin A micronutrient deficiency control interventions); therefore, the recommendation may not reflect all perspectives. An important next step would be to conduct a validation workshop with a wider and more diverse range of fortification stakeholders in Nigeria to review and revise the framework and recommendation. Second, the Nigeria example framework completed in this article was for a design decision, which is only 1 of the 5 main decision types relevant to food fortification programs. Future work should explore testing this framework for other fortification decision types at different stages of the PIP as well as in programs carried out in different contexts. Third, there are other factors beyond evidence that influence decision making in national programs (e.g., context, politics, values, and social and economic factors) (40). Although this framework can increase the systematic use of evidence or lead to a call for generating missing country-specific data, it does not directly address any other factors. Furthermore, the buy-in and capacity of the stakeholders (particularly policymakers) who would ultimately be responsible for carrying out these processes are essential if they are to be effective and sustainable over time. Moving forward, exploration into potential barriers and areas for capacity development among stakeholders in relation to the use of such decision-making frameworks and the uptake of results for decision making in fortification programs and other overlapping micronutrient interventions is needed.

The GRADE EtD framework is a practical tool that can be used by stakeholders in national food fortification programs to facilitate and document the use of evidence to inform decisions to start, strengthen, and sustain food fortification programs. Using evidence for decision making in a systematic and transparent way can improve fortification program design, delivery, and ultimately health impacts while reducing risks associated with excess micronutrient intakes.

## Supplementary Material

nzac010_Supplemental_File

## Notes

Supported by Bill & Melinda Gates Foundation grant OPP1156469 (to VMF, MNNM, MO, LMN). The funder had no role in the study design, implementation, analysis, interpretation of the data, decision to publish, or preparation of the manuscript.

Author disclosures: The authors report no conflicts of interest.

Abbreviations used: EtD, Evidence to Decision; GRADE, Grading of Recommendations Assessment, Development, and Evaluation; PIP, program impact pathway; UL, tolerable upper intake level.
