# Supplementary material for: Decisions to Start, Strengthen, and Sustain Food Fortification Programs: An Application of the Grading of Recommendations Assessment, Development, and Evaluation (GRADE) Evidence to Decision (EtD) Framework in Nigeria
Source: Curr Dev Nutr. 2022 Jan 31;6(3):nzac010. doi: 10.1093/cdn/nzac010 (PMC8894290; doi:10.1093/cdn/nzac010)
Supplement: nzac010_Supplemental_File [file nzac010_supplemental_file.docx]

**SUPPLEMENTAL FILE 1.** Evidence to decision (EtD) framework for a decision about modifying the design of Nigeria’s large-scale food fortification program to reduce the risk of excessive vitamin A intakes

**Should the design of Nigeria’s large-scale food fortification program, which aims to reduce vitamin A deficiency, be modified to reduce the risk of excessive vitamin A intakes?** (Health system and public health recommendation)

QUESTION

***Question details***

**Problem:** Reducing vitamin A deficiency through large-scale food fortification without exceeding the upper tolerable intake level (UL) for vitamin A intakes in any part of the population

**Option:** Modify the design of the fortification program by updating the selection of foods to be fortified with vitamin A and/or amounts of vitamin A to be added based on recent data on population need and consumption patterns

**Comparison:** Continue implementation of the fortification program as currently mandated (i.e., fortification of oil; sugar; and wheat, maize, and semolina flours with vitamin A as per current national standards)

**Main outcomes:**

- Vitamin A deficiency prevalence
- Vitamin A intakes from all dietary sources (i.e., fortified foods and other foods, beverages, and supplements)
- Vitamin A intakes from fortified foods alone

**Setting:** Nigeria

**Perspective:** Population-level

------------------------------------------------------------------------------------------------------------------------------

***Background***

Large-scale food fortification is proven to be a cost-effective intervention to reduce micronutrient deficiencies and their associated outcomes when programs are appropriately designed and implemented (32). In Nigeria, the prevalence of vitamin A deficiencies is high and persistent (20). Several interventions are currently in place that aim to increase vitamin A intakes in the population, including the mandatory fortification of oil, sugar, and wheat, semolina, and maize flours since 2002 (15-19).

This framework presents the research evidence and other relevant information on modifying Nigeria’s large-scale food fortification program by updating the selection of foods to be fortified with vitamin A and/or amounts of vitamin A to be added to the selected foods based on recent data on population need and consumption patterns to reduce the risk of excessive vitamin A intakes.

***Subgroups***

**Subgroup name:** Women of reproductive age (15 to 49 years)

**Subgroup name:** Children (under 5 years)

ASSESSMENT

***Problem***

Is the problem a priority?

**Judgement**

| Don't know | Varies | No | Probably No | Probably Yes | Yes |
| --- | --- | --- | --- | --- | --- |

**Research evidence**

In the most recent national micronutrient survey conducted in Nigeria in 2001, approximately 30% of children under five years had vitamin A deficiency (serum retinol concentration < 20 µg/dl) and 13% of mothers and 19% of pregnant women were at risk of vitamin A deficiency (< 30 µg/dl), of whom 4% and 9%, respectively, were deficient (<20 µg/dl) (20).

To increase vitamin A intakes in the population, several interventions are currently in place. These include mandatory fortification of five staple foods with vitamin A (i.e., oil; sugar; and wheat, semolina, and maize flours) since 2002 as well as public health supplementation, point-of-use fortification, biofortification, promotion of dietary diversity, voluntary fortification (e.g., infant formula, powdered milk, and cocoa drinks), and ad hoc individual supplement use (21).

Unfortunately, there are no more recent national data available on the prevalence of vitamin A deficiency (nor adequacy of intakes) to demonstrate the extent to which the introduction of these programs has led to reduced deficiency prevalence (or increased intakes). At the same time, concerns have been raised in Nigeria regarding the risk of potentially excessive micronutrient intakes because of these multiple overlapping interventions that target the same micronutrient and similar population groups (21). Where there are multiple interventions in place that overlap in target population groups, there is a risk of excessive micronutrient intakes if they are not coordinated and closely monitored (9,10). For preformed vitamin A specifically, the effects of chronic excessive intakes can lead to toxicity, which may cause severe adverse effects among young children and women of reproductive age are (e.g., liver damage and teratogenicity) (22). It is therefore critical to ensure that the total vitamin A intake in the diet coming from all sources does not result in intakes routinely exceeding the UL in any part of the population yet is still enough to shift inadequate intakes to adequate.

**Additional considerations**

None

***Desirable effects***

How substantial are the desirable anticipated effects?

**Judgement**

| Don't know | Varies | Trivial | Small | Moderate | Large |
| --- | --- | --- | --- | --- | --- |

**Research evidence**

There are no included randomized controlled trials, systematic reviews, or meta-analyses comparing the option (updating the selection of foods to be fortified with vitamin A and/or amounts of vitamin A to be added to the selected foods based on current population need and consumption patterns) and the comparison (continuing the vitamin A fortification program as is) for the main outcomes (vitamin A deficiency and vitamin A intakes in the diet).

However, there were relevant findings from one observational (cross-sectional) study that reported apparent vitamin A intakes from fortified foods alone based on actual consumption patterns. In that study, it was estimated that apparent vitamin A intakes would exceed the UL in 18% and 56 % of women of reproductive age in Lagos and Kanos states, respectively, if all foods were fortified with accordance with national standards (3). This analysis did not account for vitamin A intakes from other interventions; therefore, the total dietary intake from all sources is likely to be much higher in some population groups given the other vitamin A interventions being implemented (i.e., public health supplementation, point-of-use fortification, biofortification, promotion of dietary diversity, voluntary fortification, and ad hoc individual supplement use) (21).

That said, currently compliance with mandatory fortification standards in Nigeria has been shown to be consistently poor with most foods (apart from salt) being fortified below standards or not at all (11,23,24) and the coverage of other vitamin A interventions, such as supplementation and promotion of dietary diversity, is similarly low, e.g., in 2018, vitamin A supplementation reach was 41% among children 6-59 months nationally (25) and in 2013, only 52% of children 6-23 months reported having consumed vitamin A rich foods (26). This likely reduces the current risk of excessive vitamin A intakes (while increasing the risk of deficiency); however, if these programs were to improve and be implemented as intended, the risk of excessive vitamin A intakes is likely to increase.

**Additional considerations**

A systematic review and meta-analysis of vitamin A fortification programs in low- and middle-income countries confirms that vitamin A fortification is associated with improved nutritional outcomes, i.e., a significant increase in serum retinol (standard mean difference: 0.31; 95% CI: 0.18, 0.45) among children 12-24 months (4 studies, n=2800, moderate certainty of evidence) and a single study among women of reproductive age showed similar improvements (SMD: 0.45 (95% CI: 0.26, 0.64), n=447 (1).

***Undesirable effects***

How substantial are the undesirable anticipated effects?

**Judgement**

| Don't know | Varies | Large | Moderate | Small | Trivial |
| --- | --- | --- | --- | --- | --- |

**Research evidence**

No adverse effects of updating the selection of foods to fortify with vitamin A and/or amounts of vitamin A in the selected foods in the fortification program based on current population need and consumption patterns are anticipated if the redesign is done according to global guidelines on designing food fortification programs (32), assuming the redesigned program is implemented effectively.

***Certainty of the evidence***

What is the overall certainty of the evidence of effects?

**Judgement**

| No included studies | Very low | Low | Moderate | High |
| --- | --- | --- | --- | --- |

**Research evidence**

The overall certainty for the option is very low owing to the limited availability of evidence to assess, i.e., only one observational (cross-sectional) subnational study included.

However, this is not surprising given that high certainty evidence studies (e.g., randomized controlled trials, systematic reviews) examining the option vs. the comparison are not feasible nor necessary to undertake in the context of national population-based programs when efficacy of the intervention has already been demonstrated in such studies as is the case for vitamin A fortification (1).

***Values***

Is there important uncertainty about, or variability in, how much people value the main outcomes?

**Judgement**

| Important uncertainty or variability | Possibly important uncertainty or variability | Probably no important uncertainty or variability | No important uncertainty or variability |
| --- | --- | --- | --- |

**Research evidence**

There was no evidence on how the population (and specific sub-groups) value the main outcomes that were considered.

**Additional considerations**

None

***Balance of effects***

Does the balance between desirable and undesirable effects favor the option or the comparison?

**Judgement**

| Don't know | Varies | Favors the comparison | Probably favors the comparison | Does not favor either the option or the comparison | Probably favors the option | Favors the option |
| --- | --- | --- | --- | --- | --- | --- |

**Research evidence**

See the four preceding criteria.

***Resources required***

How large are the resource requirements (costs)?

**Judgement**

| Don't know | Varies | Large costs | Moderate costs | Negligible costs or savings | Moderate savings | Large savings |
| --- | --- | --- | --- | --- | --- | --- |

**Research evidence**

Costs incurred by food processors related to the fortification process itself (e.g., premix, equipment, training, human resources) and by government ministries related to ongoing monitoring and regulation at industry and market levels (e.g., analytical capacity for testing micronutrients, equipment, training, human resources) must be considered along with costs required to implement other overlapping vitamin A interventions. However, there was no available evidence to estimate costs of implementing the option nor of the current costs for implementing the comparison and the cumulative costs of all vitamin A ongoing interventions are unknown.

While food fortification programs have been proven to be among the most cost-effective interventions for improving nutrition (32), cost-effectiveness studies for the Nigeria fortification program specifically have not yet been done. Additionally, optimizing the set of vitamin A interventions that can be effectively implemented in Nigeria to achieve the greatest impact would likely reduce cost inefficiencies of running multiple programs with low fidelity (compliance).

**Additional considerations**

It is assumed that if the fortification program were modified to include a reduced number of food vehicles requiring the addition of vitamin A, there would likely be cost savings among both food processors and government ministries as fewer foods would mean some food processors would not need to fortify at all if vitamin A were the only micronutrient required to be added previously (e.g., sugar or oil) and thus monitoring efforts for those foods would no longer be needed.

Furthermore, if the vitamin A program were modified to reduce the amount of vitamin A required in the current five food vehicles or remove vitamin A from the standard for foods that still require other micronutrients to be added (e.g., flours), there may also be some cost savings on the side of the food producer (e.g., reduced premix cost) but other aspects of the fortification process would still be required as would government monitoring efforts.

Conversely, if the program were modified to include additional food vehicles requiring the addition of vitamin A, there would likely be additional costs among both food processors and government ministries for both the fortification process and monitoring efforts.

***Certainty of evidence of required resources***

What is the certainty of the evidence of resource requirements (costs)?

**Judgement**

| No included studies | Very low | Low | Moderate | High |
| --- | --- | --- | --- | --- |

**Research evidence**

No studies available.

**Additional considerations**

Costs are likely to vary across food processors and settings within Nigeria.

***Cost-effectiveness***

Does the cost-effectiveness of the option favor the option or the comparison?

**Judgement**

| Don't know | Varies | Favors the comparison | Probably favors the comparison | Does not favor either the option or the comparison | Probably favors the option | Favors the option |
| --- | --- | --- | --- | --- | --- | --- |

**Research evidence**

While there may be potential for moderate savings associated with modifying Nigeria’s vitamin A fortification program (see section on resources required), there was no evidence to estimate the resource requirements nor the cost-effectiveness evidence of the option versus the comparison.

**Additional considerations**

None

***Equity***

What would be the impact on health equity?

**Judgement**

| Don't know | Varies | Reduced | Probably reduced | Probably no impact | Probably increased | Increased |
| --- | --- | --- | --- | --- | --- | --- |

**Research evidence**

There is evidence from two cross-sectional surveys in 4 states out of the 36 in Nigeria (Kano, Lagos, Ebonyi, and Sokoto) that the coverage of fortifiable foods that are currently required to be fortified with vitamin A in Nigeria (i.e., the comparison) is generally lower in vulnerable households, specifically those that are at risk of poverty (multi-dimensional poverty index ≥0.33), have low socioeconomic status (lowest two wealth quintiles), and have low dietary diversity (women’s dietary diversity score <5) (27,28).

Similar trends have been shown in other countries and may be due to issues of access, affordability, and/or limited consumption of the respective fortified foods among these at-risk groups (11).

Comparatively, in Ebonyi and Sokoto, coverage of fortifiable bouillon (which is not currently included in the fortification program) was found to be universal (>98%) with no differences by vulnerable group (28).

While updating the selection of foods to fortify with vitamin A based on current consumption patterns would not change existing inequities related to access and affordability, the process would be able to identify which foods currently being fortified and which alternative and/or additional foods have the greatest potential to reach vulnerable populations.

**Additional considerations**

None.

***Acceptability***

Is the option acceptable to key stakeholders?

**Judgement**

| Don't know | Varies | No | Probably No | Probably Yes | Yes |
| --- | --- | --- | --- | --- | --- |

**Research evidence**

Fortification of staple foods in Nigeria is assumed to be generally accepted by the population as it does not change the characteristics of the food nor require any changes to consumption patterns, but no studies are available to confirm this.

Other key stakeholders, including policymakers, food processors, and development partners, have publicly demonstrated their support for the national fortification program in its current form while recognizing the need for improvements if reductions in micronutrient deficiencies are to be realized. For example, in 2018, industry, government, and development partners made commitments at the Nigeria Food Processing and Nutrition Leadership Forum to achieving fortification goals by 2020 (29). In 2019, the Federal Ministry of Health and partners hosted the first national micronutrient conference where they discussed the urgent need to address micronutrient deficiencies by leveraging strengths to scale up the various interventions in the country, including food fortification, supplementation, and dietary diversification (30). Additionally, in 2016, government officials delivered the Lagos statement on Nigeria Food Fortification statement at the Nigeria Future Fortified Stakeholders’ Dialogue that outlined a roadmap of activities for effectively implementing the fortification program (31).

Since these stakeholders are already accepting of the current fortification program, it is assumed that they would accept the option of a modified program that would better achieve its goal of reducing vitamin A deficiency while minimizing any risks of excessive intakes, but there is no evidence to confirm this.

**Additional considerations**

None.

***Feasibility***

Is the option feasible to implement?

**Judgement**

| Don't know | Varies | No | Probably No | Probably Yes | Yes |
| --- | --- | --- | --- | --- | --- |

**Research evidence**

Updating the selection of foods to fortify with vitamin A and/or amounts of vitamin A to add based on current population need and consumption would require recent population data on vitamin A intakes and consumption patterns of fortifiable foods. In addition, recent data on vitamin A status would be ideal to serve as a new baseline for which to evaluate impact against in the future. These data are forthcoming as part of the 2021 National Food Consumption and Micronutrient Survey (data are expected to be available in 2022).

Appropriate technical support and related funding to analyze the new data and propose specific program design changes would also be needed, which may be potential barriers in Nigeria.

Once redesigned, fortification standards would need to be updated along with any necessary legislative changes, which would require government buy-in and may be a possible barrier if stakeholders are not accepting of the option or processes are lengthy.

Finally, once approved, changes to food producer processes (either stop or start fortification or change premix) and regulatory monitoring efforts (potentially stop monitoring some foods altogether or for vitamin A) would be needed but efforts to implement such activities would likely be reduced rather than increased.

**Additional considerations**

If the option is implemented, once designed and approved by the government, it would be delivered through the existing fortification program structures as the current fortification program and thus as likely to succeed as the comparison (i.e., the current program).

CONCLUSIONS

***Summary of judgements***

| Problem | -  Don't know | -  Varies |  | -  No | -  Probably No | **✓**  Probably Yes | -  Yes |
| --- | --- | --- | --- | --- | --- | --- | --- |
| Desirable effects | -  Don't know | -  Varies |  | -  Trivial | -  Small | **✓**  Moderate | -  Large |
| Undesirable effects | -  Don't know | -  Varies |  | -  Large | -  Moderate | -  Small | **✓**  Trivial |
| Certainty of the evidence | -  No included studies |  |  | **✓**  Very low | -  Low | -  Moderate | -  High |
| Values |  |  |  | -  Important uncertainty or variability | -  Possibly important uncertainty or variability | **✓**  Probably no important uncertainty or variability | No important uncertainty or variability |
| Balance of effects | -  Don't know | -  Varies | -  Favors the comparison | -  Probably favors the comparison | -  Does not favor either the option or the comparison | **✓**  Probably favors the option | -  Favors the option |
| Resources required | **✓**  Don't know | -  Varies | -  Large costs | -  Moderate costs | -  Negligible costs or savings | Moderate savings | -  Large savings |
| Certainty of evidence of required resources | **✓**  No included studies |  |  | -  Very low | Low | -  Moderate | -  High |
| Cost-effectiveness | **✓**  Don't know | -  Varies | -  Favors the comparison | -  Probably favors the comparison | -  Does not favor either the option or the comparison | Probably favors the option | -  Favors the option |
| Equity | -  Don't know | Varies | -  Reduced | -  Probably reduced | -  Probably no impact | **✓**  Probably increased | -  Increased |
| Acceptability | **✓**  Don't know | -  Varies |  | -  No | -  Probably No | Probably Yes | -  Yes |
| Feasibility | -  Don't know | -  Varies |  | -  No | -  Probably No | **✓**  Probably Yes | Yes |

***Type of recommendation***

**Judgement**

| Strong recommendation against the option | Conditional recommendation against the option | Conditional recommendation for either the option or the comparison | Conditional recommendation for the option | Strong recommendation for the option |
| --- | --- | --- | --- | --- |

***Recommendation***

Modifying the design of Nigeria’s large-scale food fortification program to reduce the risk of excessive vitamin A intakes in the population by updating the selection of foods to be fortified with vitamin A and/or amounts of vitamin A to be added based on data on population need and consumption pattern is conditionally recommended.

***Justification***

Modifying the design of Nigeria’s large-scale food fortification program provides an opportunity to ensure it accounts for all vitamin A sources in the diet (given the high number of vitamin A interventions implemented simultaneously) and follows global guidance principles. Doing so would minimize the risk of excessive vitamin A intakes and probably increase equity.

**Detailed justification**

| Problem Desirable effects Undesirable effects Certainty of the evidence Values Balance of effects Resources required Certainty of evidence of required resources Cost-effectiveness Equity Acceptability Feasibility | The certainty of the evidence is very low.  Updating the selection of foods to fortify with vitamin A and/or the amounts of vitamin A to be added based on current data on population need and consumption patterns (accounting for all sources of vitamin A in the diet, particularly those from other vitamin A interventions being carried out) would likely reduce the risk of excessive intakes of vitamin A (while increasing adequate intakes).  Updating the selection of foods to fortify with vitamin A probably increases equity by identifying which foods have the greatest potential to reach vulnerable populations that may not be being reached with the current fortified foods. |
| --- | --- |

***Subgroup considerations***

The modifications to Nigeria’s fortification program design should be based on the demonstrated vitamin A needs and consumption patterns of fortifiable foods among different subpopulation groups (e.g., children under five years, adolescent boys and girls, women of reproductive age, and adult men) following global guidance on designing fortification programs (FAO/WHO guidelines 2006) and using the most recent data once available from the 2021 national food consumption and micronutrient survey in Nigeria.

***Implementation considerations***

- To have an impact on reducing vitamin A deficiency, there needs to be:
  - adequate compliance with national fortification standards by food processors and effective monitoring and enforcement by government to ensure high quality fortified foods are available to the population; and
  - high population coverage of fortified foods so that they are consumed in adequate amounts to making meaningful contributions to vitamin A requirements.
- Monitoring of vitamin A intakes from fortified foods must be coordinated with that from other interventions that similarly aim to increase vitamin A intakes to ensure that the total vitamin A intake in the diet is considered when reviewing risks of excessive intakes.

The political/social context:

- Political support (national and local level) is essential.
- Implementing the intervention as part of the national nutrition strategy is likely to enhance coverage and sustainability.

***Monitoring and evaluation***

- Implementation of this recommendation should be subject to ongoing monitoring to ensure high quality implementation according to its design, including:
  - compliance monitoring at import, production, and market levels with effective enforcement measures; and
  - coverage and consumption monitoring at household and individual levels.
- Evaluation of impact on reducing vitamin A deficiency in the population should not be undertaken until sufficient evidence from monitoring data is available to demonstrate that all previous stages in the program impact pathway have been achieved.

***Research priorities***

- Generate the new data on nutrient status, nutrient gaps, and food consumption patterns required to carry out this recommendation (forthcoming in the national food consumption and micronutrient survey is currently being carried out in Nigeria in 2021).
- Determine the resources required and cost-effectiveness of the option versus the comparison.
- Determine the acceptability of implementing the option among stakeholders.
